# Effects of exergaming on executive functions of children: a systematic review and meta-analysis from 2010 to 2023

**DOI:** 10.1186/s13690-023-01195-z

**Published:** 2023-10-13

**Authors:** Jiaqi Chen, Xiaojiao Zhou, Xiangting Wu, Zan Gao, Sunyue Ye

**Affiliations:** 1 Department of Preschool Education, Jiaxing University, Jiaxing, China; 2 Institute of Child Development, Jiaxing University, Jiaxing, China; 3 Department of Kinesiology, Recreation, and Sport Studies, The University of Tennessee, Knoxville, TN USA

**Keywords:** Cognitive flexibility, Exergame, Inhibition control, Physical activity, Working memory

## Abstract

**Background:**

Executive function plays a crucial role in children’s cognitive development, academic performance, as well as their physical and mental health. This study aims to assess the impact of exergaming on executive functions in pediatric populations.

**Methods:**

The criteria of inclusion were randomized controlled trials of exergaming intervention and evaluation of executive function in children aged 4–12 years. A meta-analysis was performed in databases of China National Knowledge Infrastructure (in Chinese), Wan Fang (in Chinese), Web of Science, Embase, and PubMed, from January 2010 to February 2023, following the PRISMA guidelines. Risk of bias was assessed by the Jadad scale, the Cochrane risk of bias assessment tool, funnel plot, and regression-based Egger test. The Review Manager 5.3 was used to analyze the included articles using a random-effects model, and the effects were calculated as standardized mean difference (SMD).

**Results:**

Eleven experimental studies with children (n = 508) were included. Exergaming was found to have a positive impact on children’s cognitive flexibility (SMD = 0.34, 95%CI [0.17,0.52], *P < 0.01*), inhibitory control (SMD = 0.57, 95%CI [0.31,0.83], *P < 0.01*), and working memory (SMD = 0.26, 95%CI [0.02,0.51], *P < 0.05*). The publication bias were observed.

**Conclusions:**

Exergaming has the potential to improve executive functions in children. More studies with rigorous designs are warranted to explore the specific effects of exergaming intervention. This study was registered on the PROSPERO (CRD42023401526).

**Supplementary Information:**

The online version contains supplementary material available at 10.1186/s13690-023-01195-z.

**Table:** 

**Text box 1. Contributions to the literature**
• Executive functions are cognitive processes that involve deliberate, organized, and concentrated control over complex tasks to achieve a specific goal.
• Exergaming refers to physical activity that involves playing video games and has been suggested as a potential means of enhancing the executive functions of children, though the evidence for its effectiveness is currently inconclusive.
• A meta-analysis of recent research indicates that exergaming has the potential to improve the executive functions of children, particularly those with special needs.
• This study represents the most comprehensive overview to date of the effects of exergaming programs on the executive functions of children.

## Introduction

Executive functions refer to the planned, organized, and focused control process for the goal of a complex cognitive task [1], which includes three core functions (inhibition control, working memory, and cognitive flexibility) and other higher-level functions such as reasoning, planning, and problem-solving [2]. They are a set of cognitive skills that allow children to plan, focus, remember instructions, and multitask [3]. Previous studies indicate that executive functions are essential in children’s development as they play a pivotal role in helping children learn how to regulate their behavior, think flexibly, and stay organized [4, 5]. Children can better control their behavior, follow directions, and remember important information by developing strong executive functions [6]. Executive functions can help them in their journey to success in school and life [7]. The earlier the children develop the executive function, the more conducive to their daily life and learning performance in the future [8]. Therefore, executive function is of great significance to children’s development.

Physical activity plays an important role in the development of executive functions in children [9]. Studies have shown that regular physical activity can improve children’s executive functions, such as memory, planning, and problem-solving [10, 11]. It also helps to reduce stress and anxiety, both of which can interfere with executive functions [12]. Furthermore, physical activity helps to build strong connections between neurons, which is important for developing executive functions [13]. Therefore, children need to engage in regular physical activity to improve their executive functions. Among various physical activity programs, exergaming has been increasingly studied in children and adults in recent years [14–16]. Exergaming is a video game activity based on motion-sensing technology and devices, which can read and interpret the user’s body movements and give feedback via the screen [17]. Children do not need to use traditional controllers such as a keyboard and mouse. They can participate in games through their body movements, which is easier to bring an immersive experience [18]. Research has found that exergaming can lead to improved physical fitness, motor skills [19], and even academic performance in children. Furthermore, exergaming may enhance children’s executive functions more than traditional non-exercise video games or single aerobic exercise sessions [20, 21]. Overall, the research has found that exergaming can be a beneficial activity great option for promoting children’s cognition and physical activity [22]. It is essential to provide opportunities for children to participate in this type of play.

According to the literature, engaging in exergaming might be a favorable approach to improve children’s executive functions in a fun and interactive way [22]. However, the effectiveness of exergaming on executive functions remains unclear and the findings are inconclusive [23–25]. This meta-analysis aims to examine the influence of exergaming on children’s executive function, with a focus on inhibition control, working memory, and cognitive flexibility. It quantitatively assesses the impact of exergaming on executive functions in pediatric populations, including health and special children. By synthesizing and analyzing the existing body of research, this study seeks to provide a comprehensive understanding of the effects of exergaming on children’s cognitive abilities. The findings from this meta-analysis will contribute to the growing field of exergaming research and shed light on the potential benefits of incorporating exergaming interventions in promoting children’s executive function.

## Data and methods

This meta-analysis followed the Preferred Reporting Items for Systematic Reviews and Meta-analyses protocols (PRISMA-P 2009) reporting guidelines [26]. The protocol was registered on the PROSPERO (https://www.crd.york.ac.uk/prospero/) and the registration number is CRD42023401526.

### Data source

The electronic databases of China National Knowledge Infrastructure (in Chinese), Wan Fang (in Chinese), Web of Science (in English), Embase (in English), and PubMed (in English) were searched for studies investigating the influence of exergaming on children’s executive function. The publication time of articles was from January 2010 to February 2023. The key search terms were (“exergame” OR “active video game” OR “video game”) AND “child” AND (“executive function” OR “cognitive functions” OR “inhibition control” OR “working memory” OR “cognitive flexibility”). The keywords in Chinese included “体感游戏”, “儿童”, “执行功能”, “认知灵活性”, “抑制控制”, and “工作记忆”. The list of references was also checked.

### Literature inclusion and exclusion criteria

#### Participants

Healthy children or special children aged 4–12 years. Healthy children: no previous history of neurological or psychiatric disorders. Special children: children diagnosed with autism spectrum disorder (ASD), attention deficit hyperactivity disorder (ADHD), or other disorders by clinical or parental reports.

#### Intervention

Exergaming or active video games, such as using Microsoft Kinect, or Nintendo Wii exergaming console.

#### Comparison

No intervention, conventional exercise, or others (e.g., medications)

#### Outcomes

Cognitive flexibility, inhibition control, and working memory. Cognitive flexibility refers to an individual’s ability to accurately and quickly adjust our thoughts and behaviors based on changes in the external environment and internal states [27]. Inhibition control is an active suppression process that prevents irrelevant information from entering working memory to ensure the integrity of cognitive processes [28]. Working memory is a capacity-limited memory system that temporarily processes and stores information [29]. There were no specific requirements of measures for the included literature regarding questionnaires or experimental tasks.

#### Study design

Randomized controlled trials

#### Exclusion criteria

Participants included adults only; article types were limited to review studies, dissertations, conference reports, book chapters, or policy documents; the intervention measures were either non-exergaming or unknown; outcomes did not include executive functions or their components; and data was either missing or incomplete.

### Literature screening and data extraction

After removing duplicates, all titles and abstracts were independently screened by two researchers (JC, XW) based on inclusion and exclusion criteria. The full texts of the papers were screened based on the initial screening. The opinions of the third author (SY) would be consulted if there was an inconsistency in screening until a consensus was reached. The data of included studies about sample size, mean value, and standardized difference of executive functions in experimental group and control group including pre- and post- intervention were extracted (Supplemental Table 1). When using two or more measurement tasks to evaluate the same executive function domain in a study, the result of selecting the most popular measurement task was used [30]. When there are multiple measurement scores for an executive function domain, the one that requires a higher level of executive function should be selected for meta-analysis. For example, non-perseverative errors are selected as the outcome measure in the Wisconsin Card Sorting Test [11].

### Literature quality assessment

Two researchers (JC, XW) assessed the literature quality with the Jadad scale [31] and the Cochrane risk of bias assessment tool after full-text reading. The Jadad scale is scored by evaluating the generation of random sequences, randomization hiding, blindness (“appropriate"=2, “unclear"=1, “inappropriate"=0), and withdrawal (“described = 1”, “not described"=0). A score of 0–3 is deemed low quality, and a score of 4–7 is regarded as high quality. The Cochrane library systematic review manual was divided into seven areas. The two researchers (JC, XW) independently evaluated the potential biases of the study. In disagreement, the literature risk bias was determined through a collective discussion.

### Statistical analysis

Review Manager 5.3 was used to summarize and statistically analyze the results of all included studies using a random-effects model. Standardized mean difference (SMD) was chosen as the effect size, and 95% confidence intervals (CI) were calculated. The SMD were computed by dividing the mean differences derived from the disparity between pre-and post-intervention by the standard deviation (SD) of the post-intervention in each group [32]. The SMD cutoff values of 0.2, 0.5, and 0.8 are correspond to effect sizes of small, medium, and large, respectively [33]. Heterogeneity analysis was carried out, and I² was used to assess inconsistency across studies [34]. To further investigate the effects of other variables on the results, subgroup analyses were conducted, including age, intervention participants, exercise intensity, exercise frequency, and intervention duration. The moderating variables causing heterogeneity were found and determined by subgroup analysis. Publication bias is primarily assessed by a funnel plot and regression-based Egger test. Nonparametric trim-and-fill analysis was used if publication bias existed. In addition, sensitivity analysis (excluding each article one by one) was conducted to determine whether to retain or exclude outliers. If the results were significant (*p* < 0.05), outliers were retained. A statistically considerable level was set at p < 0.05.

## Results

### Literature selection flow and result

**Fig. 1:**
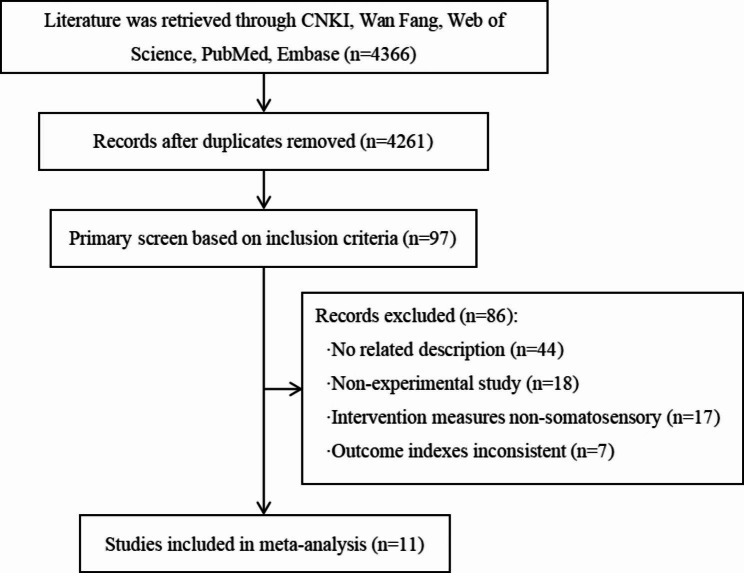
PRISMA flow diagram of the study selection

Upon searching of relevant databases, 4366 Chinese and English articles were identified; however, after eliminating duplicates, 4261 remained. After applying the inclusion and exclusion criteria, 11 papers were ultimately selected for the meta-analysis. The screening process is shown in Fig. 1.

### Basic characteristics of included studies

The sample size of this study was 508 participants aged 4 to 12 years old, with 254 belonging to the experimental group and 254 to the control group. The experimental group underwent exergaming, while the control group did conventional lifestyle or traditional sports activities. Further details can be found in Table 1.

**Table 1:** The characteristics of included studies for meta-analysis from 2010 to 2023

Studies	Participants					Intervention				Outcome indicator
	N (Int./con.)	Age, gender	Countries	Disorders		Measures	Cycle (week)	Frequency (times/week)	Duration (min/time)	
Dovis 2015 [36]	61 (31/30)	8–12, 80% male	Netherlands	ADHD		The experimental group: A specific genre of exergaming for “Brain game Brian” (BGB). The control group: Exergaming without working memory engagement.	5	/	35–50	Cognitive flexibility: TMT Inhibition control: Stop task. Working memory: CBTT
Flynn 2018 [39]	76 (35/41)	7–12, 49% male	USA	Normal		The experimental group: A specific genre of exergaming for Nintendo dance game “Hottest Door Party 2”. The control group: Non-exergaming.	1	1	60	Cognitive flexibility: Flanker task Inhibition control: flanking fish Working memory: Rule shift test
Benzing 2018 [38]	46 (24/22)	8–12, 82% male	Switzerland	ADHD		The experimental group: A specific genre of exergaming for XBOX Kinect “Shape Up”. The control group: Non-exergaming.	1	1	15	Cognitive flexibility: Flanker task Inhibition control: Simon task Working memory: CSBT
Benzing 2019 [35]	44 (22/22)	8–12, 82% male	Switzerland	ADHD		The experimental group: A specific genre of exergaming for XBOX Kinect “Shape Up”. The control group: non-exergaming.	8	3	≥ 30	Cognitive flexibility: Flanker task Inhibition control: Simon task Working memory: CSBT
Xiong 2019 [21]	60 (30/30)	4–5, 50% male	China	Normal		The experimental group: Any genre of exergaming for Wii sports. The control group: Traditional physical activity.	8	5	20	Cognitive flexibility: DCCS
Gao 2019 [20]	32 (18/14)	4–6, 50% male	USA	Normal		The experimental group: Exergaming of Leap TV (dance and PE). The control group: Non-exergaming.	12	5	30	Cognitive flexibility: DCCS
Fronza 2020 [40]	48 (25/23)	8–10, 50% male	USA	Normal		The experimental group: Exergaming 1 (Athletics and football) exergaming2 (Skiing, tennis and darts). The control group: Non-exergaming.	9	2	20–30	Cognitive flexibility: Track test part
Rafiei Milajerdi 2021 [37]	37 (17/20)	6–10, 83% male	Iran	ASD		The experimental group: A specific genre of exergaming for XBOX Kinect tennis. The control group: Non-exergaming.	8	3	35	Cognitive flexibility: WCST
Liu 2022 [41]	48 (24/24)	4–5, 48% male	China	Normal		The experimental group: Exergaming of Just Dance. The control group: Non-exergaming.	4	5	30	cognitive flexibility: DCCS inhibition control: Go/No-Go task. working memory: Mr. Ant task
Chang 2022 [42]	32 (16/16)	6–13, 81% male	China	ADHD		The experimental group: Exergaming of Wii sports. The control group: Non-exergaming.	12	3	60	cognitive flexibility: WCST Inhibition control: Stroop task
Nekar 2022 [43]	24 (12/12)	6–18, 92% male	Korea	ASD		The experimental group: A specific genre of exergaming for UINCARE. The control group: Non-exergaming.	4	2	15	Cognitive flexibility: WCST Inhibition control: Stroop task

ASD: autism spectrum disorder; ADHD: attention deficit hyperactivity disorder; DCCS: Dimension Change Card Sorting Task; TMT: Trail Making Test; CSBT: Color Span Backwards Task; WCST: Wisconsin Card Sorting Test; CBTT: Corsi Block Tapping Task

### Risk of bias results assessment

#### Jadad scale assessment

5 papers scored 7, 1 paper scored 6, 2 papers scored 4, 2 papers scored 2, and 1 paper scored 1.

#### Cochrane risk of bias assessment

**Fig. 2:**
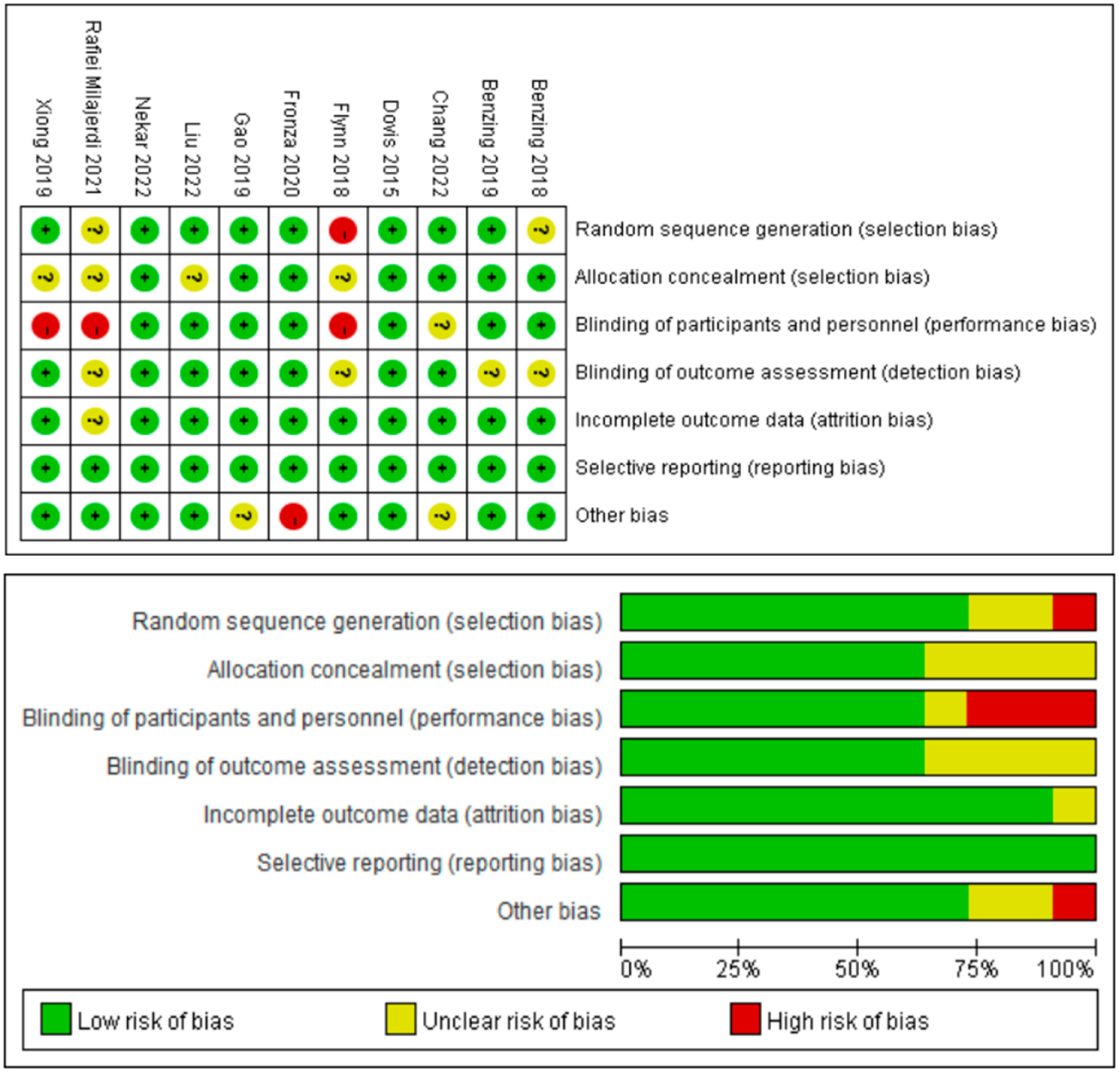
Cochrane library risk bias of exergaming on executive functions in children (2010–2023)

Eleven studies employed experimental methods, two of which did not mention the use of randomization. Seven studies described hidden allocation schemes, while the other seven were double-blind. Even though all studies experienced a loss of follow-up with some participants, they all provided explanations and processed the data accordingly as depicted in Fig. 2.

#### Publication Bias Test

**Fig. 3:**
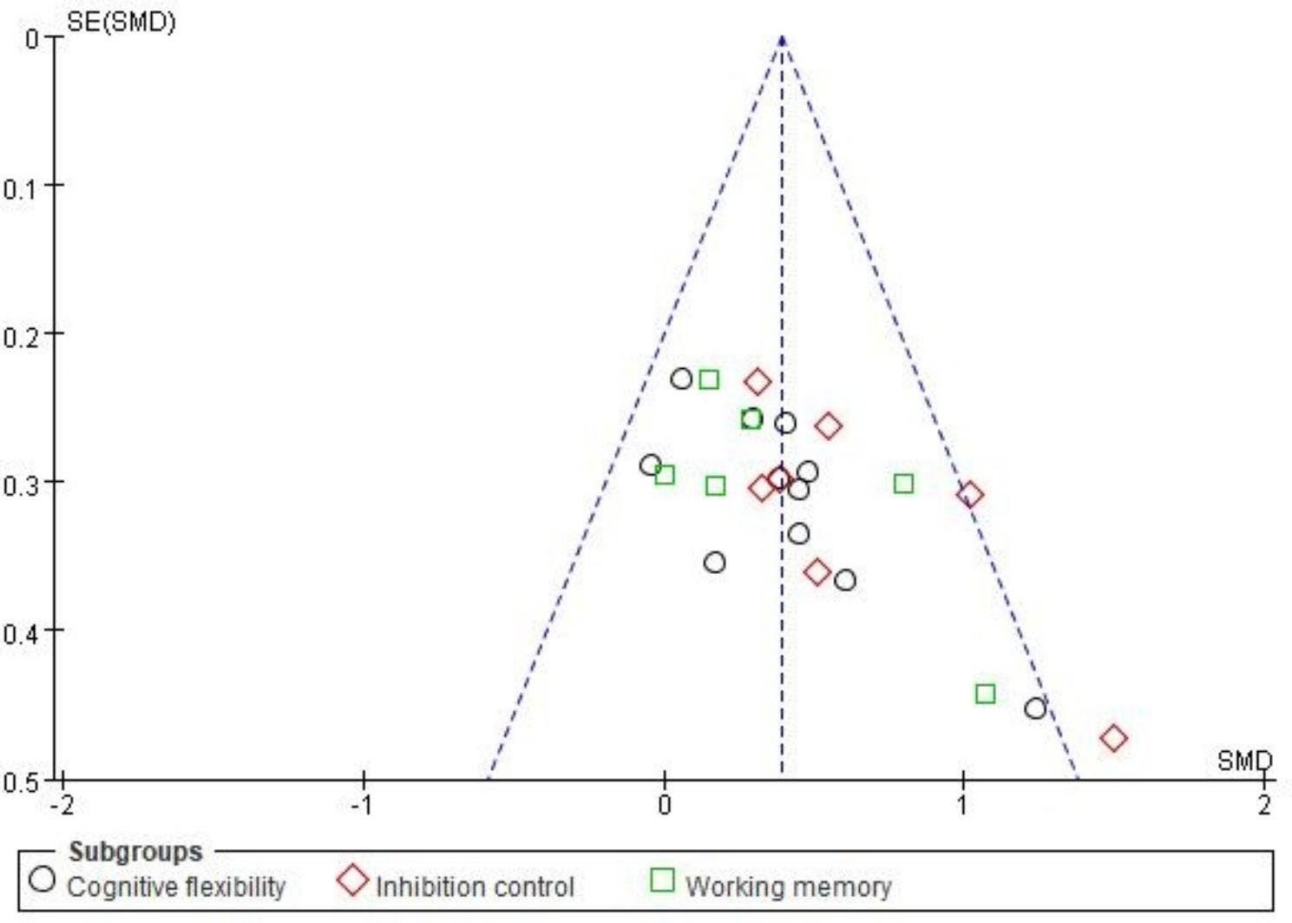
Funnel plot publication bias of exergaming and executive functions in children (2010–2023)

The funnel plot of the publication bias test including three components of executive function is shown in Fig. 3 and for subgroup analysis is shown in Supplemental Fig. 1. The results of regression-based Egger test are *p* = 0.035 for cognitive flexibility, *p* = 0.036 for inhibition control, and *p* = 0.578 for working memory, respectively.

### Integration of research evidence

**Fig. 4:**
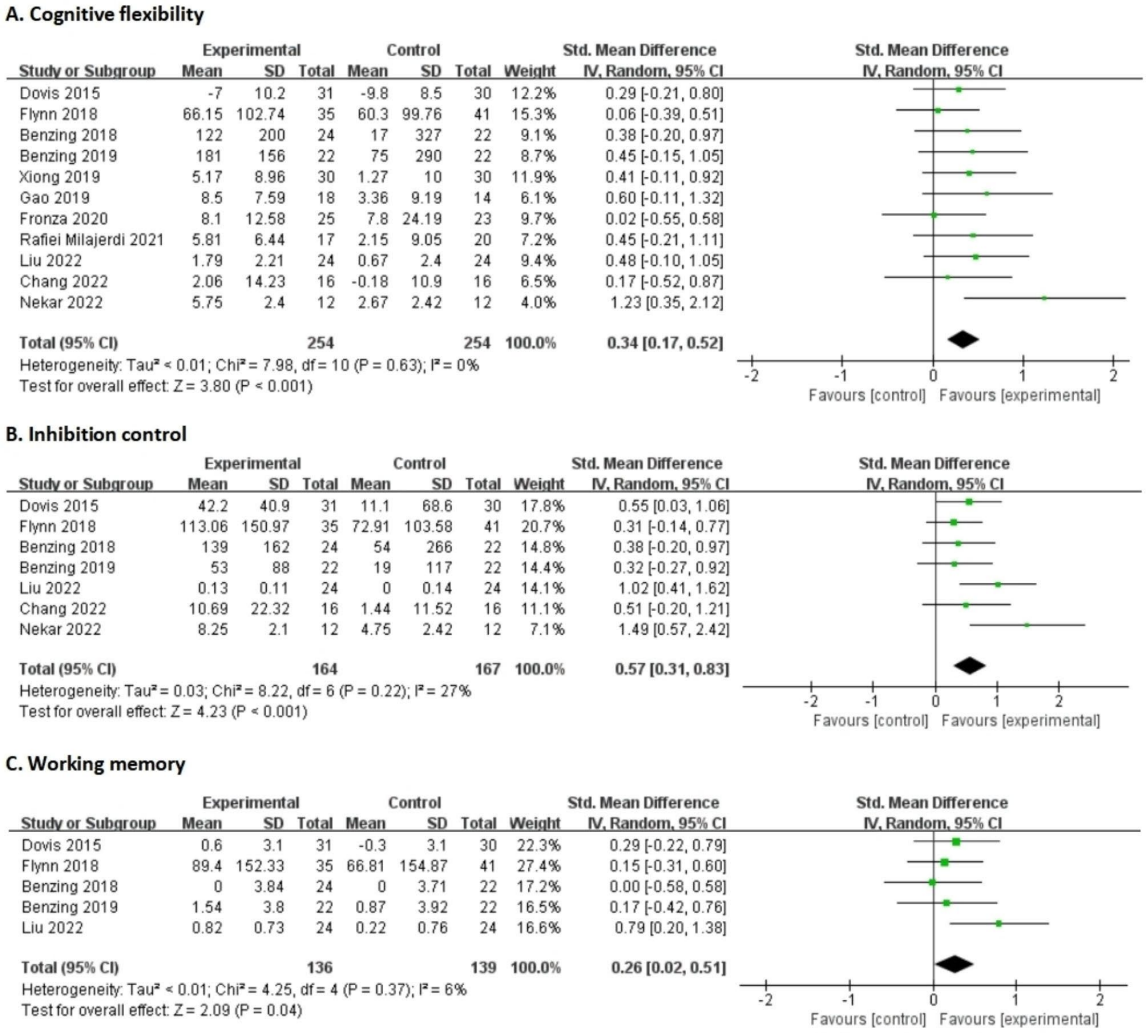
Forest plot for meta-analysis regarding the effects of exergaming intervention on children’s cognitive flexibility (A), inhibition control (B) and working memory (C) from 2010 to 2023

A total of 11 papers were included in the meta-analysis on cognitive flexibility. The results showed that SMD = 0.34, 95%CI: 0.17–0.52, *p* < 0.001, as shown in Fig. 4. The results of analysis on inhibition control and working memory showed that SMD = 0.57, 95%CI: 0.31–0.83, *p* < 0.001 and SMD = 0.26, 95%CI: 0.02–0.51, *p* < 0.05. No significant heterogeneity (I^2^ < 50%, *p* > 0.05) was observed in meta-analysis on executive functions. Sensitivity analysis shown that no significant changes were observed after excluding studies (e.g., Nekar et al., 2022) that may cause the heterogeneity. Nonparametric trim-and-fill analysis shown that SMD = 0.28, 95%CI: 0.11–0.44 for cognitive flexibility (imputed = 2), no changes for inhibition control and working memory.

In all aged subgroups of children, the improvement effects of exergaming on executive functions were statistically significant (*p* < 0.01), but not for working memory in children aged > 6 years (*p* > 0.05), See Table 2. The effects of exergaming on cognitive flexibility (in both healthy and special children) and inhibition control in special children were statistically significant (*p* < 0.05). All levels of exercise-intensity exergaming had statistically significant effects (*p* < 0.05) on children’s executive functions except for working memory. The study showed that children’s cognitive flexibility was significantly improved when exposed to 15–20 min of the activity (*p* < 0.01). Furthermore, children’s inhibition control and working memory improved when exposed to 20–30 min of the action (*p* < 0.01). Finally, cognitive flexibility and inhibition control were improved when exposed to more than 30 min of the action (*p* < 0.05). The effect was significant for children’s cognitive flexibility (*p* < 0.05) when the intervention was over 6 weeks. The significant effects (*p* < 0.01) on cognitive flexibility, inhibition control, and working memory were observed in subgroups of 2–6 weeks, but not in those ≤ 1 week.

**Table 2:** Subgroup meta-analytic results of the effects for exergaming on executive functions (2010–2023)

Variables	Subgroup category	Cognitive flexibility								Inhibition control								Working memory						
		Studies	Sample size	SMD	95%CI	I^2^	T^2^	P		Studies	Sample size	SMD	95%CI	I^2^	T^2^	P		Studies	Sample size	SMD	95%CI	I^2^	T^2^	P
Age	4–6 years	3	140	0.47	0.14, 0.81	0%	< 0.01	< 0.01		1	48	1.02	0.41, 1.62	N/A	N/A	< 0.01		1	48	0.79	0.20, 1.38	N/A	N/A	< 0.01
	> 6 years	8	368	0.29	0.09, 0.50	0%	< 0.01	< 0.01		6	283	0.48	0.23, 0.74	10%	0.01	< 0.01		4	227	0.16	-0.10, 0.42	0%	< 0.01	0.23
Disorder status	Normal	5	264	0.27	0.02, 0.51	0%	< 0.01	0.03		2	124	0.63	-0.05, 1.32	70%	0.17	0.07		2	124	0.44	-0.19, 1.07	66%	0.14	0.17
	Special	6	244	0.42	0.17, 0.68	0%	< 0.01	< 0.01		5	207	0.55	0.24, 0.86	18%	0.02	< 0.01		3	151	0.16	-0.16, 0.48	0%	< 0.01	0.31
Exercise intensity	Moderate	6	287	0.38	0.14, 0.63	7%	0.01	< 0.01		4	179	0.77	0.35, 1.19	44%	0.08	< 0.01		3	155	0.35	-0.08, 0.79	45%	0.07	0.11
	Medium-high	5	221	0.29	0.03, 0.56	0%	< 0.01	0.03		3	152	0.36	0.03, 0.68	0%	< 0.01	0.03		2	120	0.15	-0.20, 0.51	0%	< 0.01	0.40
Intervention duration	15–20 min	3	130	0.56	0.12, 1.00	31%	0.05	< 0.01		2	70	0.88	-0.20, 1.96	75%	0.46	0.11		1	46	0.00	-0.58, 0.58	N/A	N/A	1.00
	20–30 min	3	133	0.30	-0.04, 0.64	0%	< 0.01	0.09		1	48	1.02	0.41, 1.62	N/A	N/A	< 0.01		1	48	0.79	0.20, 1.38	N/A	N/A	< 0.01
	> 30 min	5	245	0.27	0.02, 0.52	0%	< 0.01	0.04		4	213	0.41	0.14, 0.68	0%	< 0.01	< 0.01		3	181	0.20	-0.09, 0.49	0%	< 0.01	0.18
Intervention cycle	≤ 1 week	2	122	0.18	-0.18, 0.54	0%	< 0.01	0.33		2	122	0.34	-0.02, 0.70	0%	< 0.01	0.06		2	44	0.09	-0.27, 0.45	0%	< 0.01	0.62
	2–6 weeks	3	133	0.56	0.09, 1.02	39%	0.07	0.02		3	133	0.92	0.42, 1.41	43%	0.08	< 0.01		2	109	0.52	0.02, 1.01	39%	0.05	0.04
	> 6 weeks	6	253	0.34	0.09, 0.58	0%	< 0.01	< 0.01		2	76	0.40	-0.06, 0.85	0%	< 0.01	0.09		1	151	0.17	-0.42, 0.76	N/A	N/A	0.57

SMD, standardized mean difference

## Discussion

This meta-analysis provides an overview of the current research on the impact of exergaming on children’s executive functions. Its main objective is to review and synthesize the findings of recent experimental exergaming studies. Eleven studies met the inclusion criteria and were included in our final analysis [20, 21, 35–43]. Overall, our data suggested that exergaming interventions could significantly improve executive functions (e.g., cognitive flexibility, inhibition control, and working memory) in healthy and special children aged 4–12 years old. Findings indicated significant improvements, yet more than one-quarter of the studies was deemed at risk for bias, particularly regarding performance bias.

Our sub-population analysis results show that the efficacy of exergaming on special children appears to be more consistent than that of healthy, typical children. This might be because special child typically has a lower initial level of executive function [44, 45], so they may experience more significant gains from an exergaming intervention than healthy children. For example, due to the late development of the frontal cortex in children with ASD and ADHD, the activity in the cerebellum and prefrontal lobes is weaker, leading to more inadequate executive functions [46]. Meanwhile, exercise may enhance children’s co-activation between the cerebellum and the dorsolateral prefrontal cortex [47]. Previous studies have suggested that those with lower starting cognition performance have more significant potential for enhancement, whereas those with higher starting performance have limited chances for further optimization [48]. Thus, the degree of improvement may be relatively small for healthy children due to the ceiling effect.

In this study, the findings indicated that exercising through exergaming at moderate intensity may provide better benefits for enhancing executive functions in children compared to exercising at a medium-high intensity. Previous meta-analysis studies found that engaging in physical activity at a moderate intensity level showed an improvement in executive function that was greater than when physical activity was done at a vigorous intensity [47, 49]. Another study in long-term exercise training found that, under the same mental state, the group that exercised at a higher intensity saw greater benefits in terms of improved executive functions than the group that exercised at a lower intensity [21]. Thus, further research is necessary to gain greater clarity on this topic.

Regarding the effects of intervention duration and frequency, our findings indicated that children’s executive functions were not improved by exergaming with a session time of fewer than 20 min or a frequency of no more than one week, except for cognitive flexibility. This may be due to the shorter intervention time or duration that only generates short-term and less significant improvements. Previous research has also suggested that longer intervention periods were more effective, as longer-term interventions might have longer-lasting effects on neurodevelopment and plasticity, leading to more significant improvements in executive function [35].

The current review did not have observed significant difference on executive functions between the exergaming and traditional physical activity programs due to the limited number of studies (for cognitive flexibility, n = 6, SMD = 0.22(-0.01, 0.46), *p* = 0.07; for inhibition control, n = 3, SMD = 0.35(-0.29, 0.99), *p* = 0.29; and for working memory, n = 2, SMD = 0.32(-0.57, 1.21), *p* = 0.48). Previous study found that exergaming were more effective than traditional physical activity in promoting children’s motor skill development, particularly in terms of postural stability after comparing the effects of exergaming and traditional physical activity on children’s health outcomes [50]. Xiong et al. found that exergaming also had a greater impact on children’s executive function and perceived social competence compared to traditional physical activity [21]. However, other studies have shown that both exergame and traditional physical activity or gameplay had positive effects on children’s basic movement skills and the differences between them were not significant [51, 52]. It is important to note that above studies have limitations, such as small sample sizes or short intervention periods, and therefore, more high-quality research is needed to fully understand the potential benefits and drawbacks of different types of activities.

This review’s major strength is that it provides the first comprehensive overview of the latest research on how exergaming programs affect the executive functions of healthy and special needs children. However, this study has a few limitations: First, the investigation into the effects of exergaming on children’s executive function is limited, which means that the potential moderating variables such as intervention intensity, frequency and duration have not been able to be adequately looked at. Second, publication bias were observed, while studies with null or non-significant results may remain unpublished or go unnoticed. Third, the measures of executive functions were variable, which may contribute to the measurement bias. Standardizing the measurement tools utilized in future studies could also help to improve the comparability and reliability of the results.

## Conclusions

The current experimental evidence has demonstrated that exergaming can positively affect children’s inhibition control, working memory, and cognitive flexibility. Nevertheless, more randomized controlled studies with standardized evaluation methods and processes are necessary to provide more accurate evidence of the potential benefits of exergaming to promote children’s cognition.

### Electronic supplementary material

Below is the link to the electronic supplementary material.

Supplementary Material 1: Supplemental Table 1 The mean and standardized difference (SD) of executive functions in experimental group and control group including pre- and post- intervention

Supplementary Material 2: Supplemental Fig. 1 Funnel plot publication bias of subgroup analysis

## Data Availability

The datasets used and/or analyzed during the current study available from the corresponding author on reasonable request.

## Abbreviations

ASD: Autism Spectrum Disorder
ADHD: Attention Deficit Hyperactivity Disorder
SMD: Standardized Mean Difference
CI: Confidence Intervals
DCCS: Dimension Change Card Sorting Task
TMT: Trail Making Test
CSBT: Color Span Backwards Task
WCST: Wisconsin Card Sorting Test
CBTT: Corsi Block Tapping Task.

## References

[1] Donnelly JE, Hillman CH, Castelli D, Etnier JL, Lee S, Tomporowski P, Lambourne K, Szabo-Reed AN (2016). Physical activity, fitness, cognitive function, and academic achievement in children: a systematic review. Med Sci Sports Exerc.

[2] Diamond A (2013). Executive functions. Annu Rev Psychol.

[3] Diamond A (2020). Executive functions. Handb Clin Neurol.

[4] Doebel S (2020). Rethinking executive function and its development. Perspect Psychol Sci.

[5] Shaheen S (2014). How child’s play impacts executive function–related behaviors. Appl Neuropsychol Child.

[6] Diamond A, Lee K (2011). Interventions shown to Aid executive function development in children 4 to 12 Years Old. Science.

[7] Blair C (2016). Executive function and early childhood education. Curr Opin Behav Sci.

[8] Gathercole SE, Pickering SJ, Ambridge B, Wearing H (2004). The structure of working memory from 4 to 15 years of age. Dev Psychol.

[9] Gao Z, Chen S, Sun H, Wen X, Xiang P. Physical Activity in Children’s Health and Cognition. *Biomed Res Int* 2018, 2018:8542403. 10.1155/2018/8542403 PMC6036844 30046612

[10] de Greeff JW, Bosker RJ, Oosterlaan J, Visscher C, Hartman E (2018). Effects of physical activity on executive functions, attention and academic performance in preadolescent children: a meta-analysis. J Sci Med Sport.

[11] Liang X, Li R, Wong SHS, Sum RKW, Sit CHP (2021). The impact of exercise interventions concerning executive functions of children and adolescents with attention-deficit/hyperactive disorder: a systematic review and meta-analysis. Int J Behav Nutr Phys Act.

[12] Pratt M (2021). What’s new in the 2020 World Health Organization Guidelines on physical activity and sedentary behavior?. J Sport Health Sci.

[13] Alvarez-Bueno C, Pesce C, Cavero-Redondo I, Sanchez-Lopez M, Martinez-Hortelano JA, Martinez-Vizcaino V (2017). The effect of physical activity interventions on children’s cognition and metacognition: a systematic review and Meta-analysis. J Am Acad Child Adolesc Psychiatry.

[14] Gao Z, Xiang P (2014). Effects of exergaming based exercise on urban children’s physical activity participation and body composition. J Phys Act Health.

[15] Pasco D, Roure C (2022). Situational interest impacts college students’ physical activity in a design-based bike exergame. J Sport Health Sci.

[16] Sousa CV, Hwang J, Cabrera-Perez R, Fernandez A, Misawa A, Newhook K, Lu AS (2022). Active video games in fully immersive virtual reality elicit moderate-to-vigorous physical activity and improve cognitive performance in sedentary college students. J Sport Health Sci.

[17] Ai-bo QC ZHU, Xiao-long YU, DAI (2014). Chen-chen: somatic sense interactive technology and its application in Motor Rehabilitation. Chin J Rehabil Theory Pract.

[18] Yun (2016). JL-z: Effects of Motion Sensing Games on children with autism. Chin J Clin Psychol.

[19] Ye SY, Lee JE, Stodden DF, Gao Z. Impact of Exergaming on Children’s Motor Skill Competence and Health-Related Fitness: A Quasi-Experimental Study. J Clin Med 2018, 7(9). 10.3390/jcm7090261 PMC6162827 30205483

[20] Gao Z, Lee JE, Zeng N, Pope ZC, Zhang Y, Li X (2019). Home-Based exergaming on Preschoolers’ Energy Expenditure, Cardiovascular Fitness, Body Mass Index and Cognitive Flexibility: a Randomized Controlled Trial. J Clin Med.

[21] Xiong S, Zhang P, Gao Z (2019). Effects of Exergaming on Preschoolers’ executive functions and perceived competence: a pilot randomized Trial. J Clin Med.

[22] Zeng N, Lee JE, Gao Z (2023). Effects of home-based exergaming on preschool children’s cognition, sedentary behavior, and physical activity: a randomized crossover trial. Brain Behav Immun Integr.

[23] Maillot P, Perrot A, Hartley A (2012). Effects of interactive physical-activity video-game training on physical and cognitive function in older adults. Psychol Aging.

[24] Sala G, Tatldil S, Gobet F et al. Still No Evidence That Exergames Improve Cognitive Ability: A Commentary on Stanmore. (2017). *Neuroscience & Biobehavioral Reviews* 2019, In press. 10.1016/j.neubiorev.2019.11.015 31760047

[25] Stanmore E, Stubbs B, Vancampfort D, De Bruin ED, Firth J (2017). The effect of active video games on cognitive functioning in clinical and non-clinical populations: a meta-analysis of randomized controlled trials. Neurosci Biobehavioral Reviews.

[26] Moher D, Liberati A, Tetzlaff J, Altman DG, Group P (2009). Preferred reporting items for systematic reviews and meta-analyses: the PRISMA statement. PLoS Med.

[27] Miyake A, Friedman NP, Emerson MJ, Witzki AH, Howerter A, Wager TD. The Unity and Diversity of Executive Functions and their contributions to Complex Frontal Lobe Tasks: a latent variable analysis. Cogn Psychol 2000. 10.1006/cogp.1999.0734 10945922

[28] Moutier S, Angeard N, Houde O (2002). Deductive reasoning and matching-bias inhibition training: evidence from a debiasing paradigm. Think Reasoning.

[29] Medin DL. Psychology of Learning and Motivation,28: The Psychology of Learning and Motivation 56; 2015.

[30] Xue Y, Yang Y, Huang T. Effects of chronic exercise interventions on executive function among children and adolescents: a systematic review with meta-analysis. Brit J Sport Med 2019. 10.1136/bjsports-2018-099825 30737201

[31] Jadad AR, Moore RA, Carroll D, Jenkinson C, Reynolds DJ, Gavaghan DJ, McQuay HJ (1996). Assessing the quality of reports of randomized clinical trials: is blinding necessary?. Control Clin Trials.

[32] Welsch L, Alliott O, Kelly P, Fawkner S, Booth J, Niven A (2021). The effect of physical activity interventions on executive functions in children with ADHD: a systematic review and meta-analysis. Ment Health Phys Act.

[33] Hedges LV, Olkin I (1985). Statistical methods for meta-analysis. New Dir Program Evaluation.

[34] Higgins J, Thompson SG, Decks JJ, Altman DG (2003). Measuring inconsistency in meta-analyses. Brit Med J.

[35] Benzing V, Schmidt M (2019). The effect of exergaming on executive functions in children with ADHD: a randomized clinical trial. Scand J Med Sci Sports.

[36] Dovis S, Van der Oord S, Wiers RW, Prins PJ (2015). Improving executive functioning in children with ADHD: training multiple executive functions within the context of a computer game. A randomized double-blind placebo controlled trial. PLoS ONE.

[37] Rafiei Milajerdi H, Sheikh M, Najafabadi MG, Saghaei B, Naghdi N, Dewey D (2021). The Effects of Physical Activity and Exergaming on Motor Skills and executive functions in children with Autism Spectrum Disorder. Games Health J.

[38] Benzing V, Chang YK, Schmidt M (2018). Acute Physical Activity enhances executive functions in children with ADHD. Sci Rep.

[39] Flynn RM, Richert RA (2018). Cognitive, not physical, engagement in video gaming influences executive functioning. J Cogn Dev.

[40] Fronza FC, Ferrari EP, Freitas KTD, Cardoso FL (2020). Intervention using Exergames: Effects on the executive functions of school-aged children. ETD- Educação Temática Digital.

[41] Liu ZM, Chen CQ, Fan XL, Lin CC, Ye XD (2022). Usability and Effects of a combined physical and cognitive intervention based on active Video Games for Preschool Children. Int J Environ Res Public Health.

[42] Chang SH, SJJ, Yu NY (2022). Enhancing executive functions and handwriting with a Concentrative Coordination Exercise in children with ADHD: a Randomized Clinical Trial. Percept Motor Skill.

[43] Nekar DM, Lee DY, Hong JH, Kim JS, Kim SG, Seo YG, Yu JH (2022). Effects of augmented reality game-based cognitive-motor training on restricted and repetitive behaviors and executive function in patients with Autism Spectrum Disorder. Healthcare.

[44] Shuai L, Wang Y, Li W, Wilson A, Wang S, Chen R, Zhang J (2021). Executive function training for Preschool Children with ADHD: a Randomized Controlled Trial. J Atten Disord.

[45] Demetriou EA, Lampit A, Quintana DS, Naismith SL, Song YJC, Pye JE, Hickie I, Guastella AJ (2018). Autism spectrum disorders: a meta-analysis of executive function. Mol Psychiatry.

[46] Close AD (2000). Interrelation of motor development and cognitive development and of the cerebellum and prefrontal cortex. Child Dev.

[47] McMorris T, Hale BJ (2012). Differential effects of differing intensities of acute exercise on speed and accuracy of cognition: a meta-analytical investigation. Brain Cogn.

[48] Diamond A (2012). Activities and programs that improve children’s executive functions. Curr Dir Psychol Sci.

[49] Bo Y (2021). Meta Analysis of Exercise on executive function of children with ADHD. China Sport Science and Technology.

[50] Sheehan DP, Katz L (2013). The effects of a daily, 6-week exergaming curriculum on balance in fourth grade children. J Sport Health Sci.

[51] Vernadakis N, Papastergiou M, Zetou E, Antoniou P (2015). The impact of an exergame-based intervention on children’s fundamental motor skills. Comput Educ.

[52] Mcgann J, Issartel J, Hederman L, Conlan O. Hop.Skip.Jump.Games: The effect of principled exergameplay on children’s locomotor skill acquisition. Brit J Educ Technol 2020, 51.

