# Supplementary material for: Effects of exergaming on executive functions of children: a systematic review and meta-analysis from 2010 to 2023
Source: Arch Public Health. 2023 Oct 13;81:182. doi: 10.1186/s13690-023-01195-z (PMC10571260; doi:10.1186/s13690-023-01195-z)
Supplement: Supplementary file 1 — Supplementary Material 1: Supplemental Table 1 The mean and standardized difference (SD) of executive functions in experimental group and control group including pre- and post- intervention [file 13690_2023_1195_MOESM1_ESM.docx]

Supplemental Table 1 The mean and standardized difference (SD) of executive functions in experimental group and control group including pre- and post- intervention

| Studies | Executive functions | Experimental group (Mean, SD) | |  | Control group (Mean, SD) | |
| --- | --- | --- | --- | --- | --- | --- |
|  |  | Pre-intervention | Post-intervention |  | Pre-intervention | Post-intervention |
| Dovis 2015^[36]^ | Cognitive flexibility | 58.80(10.50) | 51.80(10.20) |  | 64.40(9.60) | 54.80(8.50) |
|  | Inhibition control | 189.60(43.70) | 147.40(40.90) |  | 200.10(73.80) | 189.00(68.60) |
|  | Working memory | 9.30(2.90) | 9.90(3.10) |  | 9.80(2.60) | 9.50(3.10) |
| Flynn 2018^[39]^ | Cognitive flexibility | 944.42(99.34) | 878.27(113.85) |  | 905.01(104.11) | 844.71(116.27) |
|  | Inhibition control | 760.50(162.23) | 647.44(115.33) |  | 723.62(162.60) | 650.72(133.18) |
|  | Working memory | 784.81(172.03) | 695.41(140.89) |  | 733.30(189.72) | 666.49(165.05) |
| Benzing 2018^[38]^ | Cognitive flexibility | 1031.00(276.00) | 909.00 (200.00) |  | 1083.00(303.00) | 1066.00(327.00) |
|  | Inhibition control | 981.00(263.00) | 842.00(162.00) |  | 1013.00(319.00) | 959.00(266.00) |
|  | Working memory | 14.00(2.84) | 14.00(3.84) |  | 14.00(4.14) | 14.00(3.71) |
| Benzing 2019^[35]^ | Cognitive flexibility | 1055.00(291.00) | 874.00(156.00) |  | 1077.00(292.00) | 1002.00(290.00) |
|  | Inhibition control | 590.00(99.00) | 537.00(88.00) |  | 626.00(107.00) | 607.00(117.00) |
|  | Working memory | 14.00(2.73) | 15.54(3.80) |  | 13.57(3.61) | 14.44(3.92) |
| Xiong 2019^[21]^ | Cognitive flexibility | 48.23(7.96) | 53.40(8.96) |  | 47.33(10.69) | 48.60(10.00) |
| Gao 2019^[20]^ | Cognitive flexibility | 50.89(7.70) | 59.39(7.59) |  | 59.00(7.65) | 62.36(9.19) |
| Fronza 2020^[40]^ | Cognitive flexibility | 105.80(16.20) | 113.90(12.58) |  | 106.60(19.40) | 114.40(24.19) |
| Rafiei Milajerdi 2021^[37]^ | Cognitive flexibility | 19.11(8.00) | 13.30(6.44) |  | 21.15(7.42) | 19.00(9.05) |
| Liu 2022^[41]^ | Cognitive flexibility | 5.13(2.94) | 6.92(2.21) |  | 5.21(3.15) | 5.88(2.40) |
|  | Inhibition control | 0.71(0.16) | 0.84(0.11) |  | 0.74(0.14) | 0.74(0.14) |
|  | Working memory | 1.71(0.77) | 2.53(0.73) |  | 1.68(0.76) | 1.90(0.76) |
| Chang 2022^[42]^ | Cognitive flexibility | 22.94(14.64) | 20.88(14.23) |  | 21.63(5.67) | 21.81(10.90) |
|  | Inhibition control | 63.43(17.85) | 74.12(22.32) |  | 64.56(8.43) | 66.00(11.52) |
| Nekar 2022^[43]^ | Cognitive flexibility | 79.08(3.02) | 84.83(2.40) |  | 79.25(4.11) | 81.92(2.42) |
|  | Inhibition control | 76.08(2.06) | 84.33(2.10) |  | 76.33(3.79) | 81.08(2.42) |
