# Supplementary material for: Effects of exergaming on executive functions of children: a systematic review and meta-analysis from 2010 to 2023
Source: Arch Public Health. 2023 Oct 13;81:182. doi: 10.1186/s13690-023-01195-z (PMC10571260; doi:10.1186/s13690-023-01195-z)
Supplement: Supplementary file 2 — Supplementary Material 2: Supplemental Fig. 1 Funnel plot publication bias of subgroup analysis [file 13690_2023_1195_MOESM2_ESM.docx]

#### [Cognitive flexibility](javascript:;)：


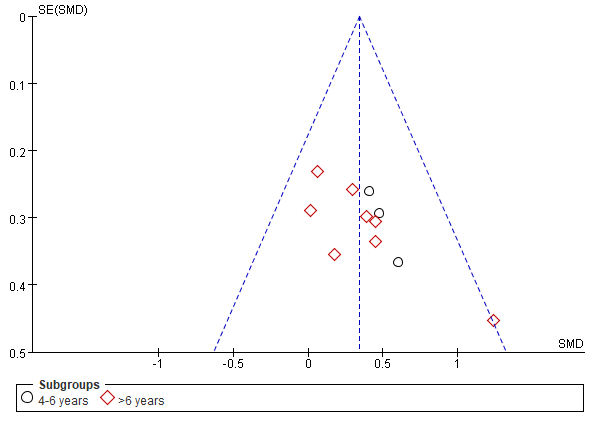


Age


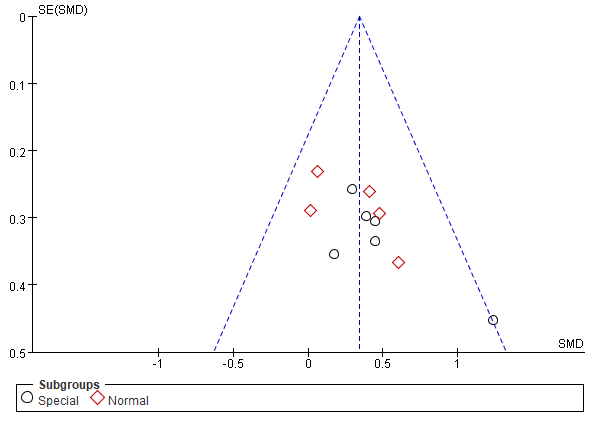


Disorder status


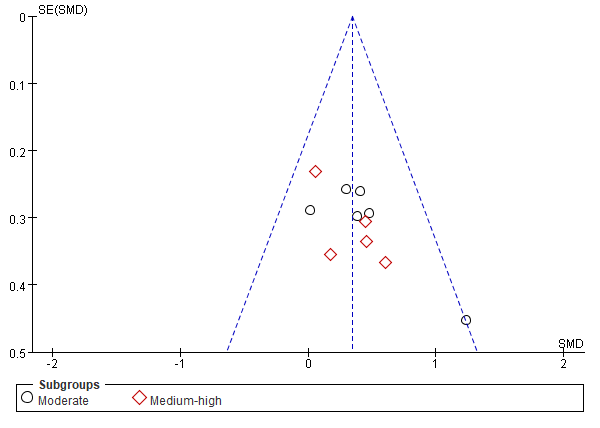


Exercise intensity


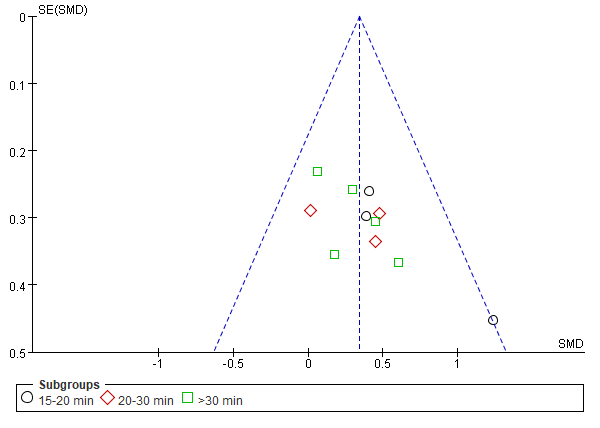


Intervention duration


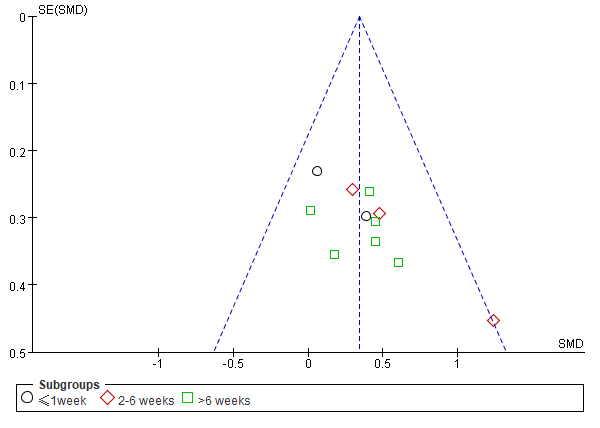


Intervention cycle

#### Inhibition control：


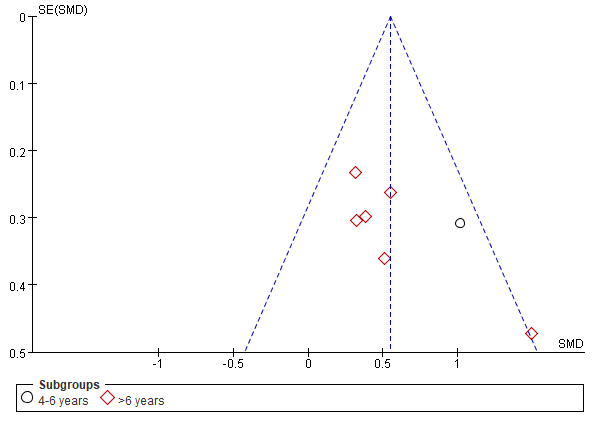


Age


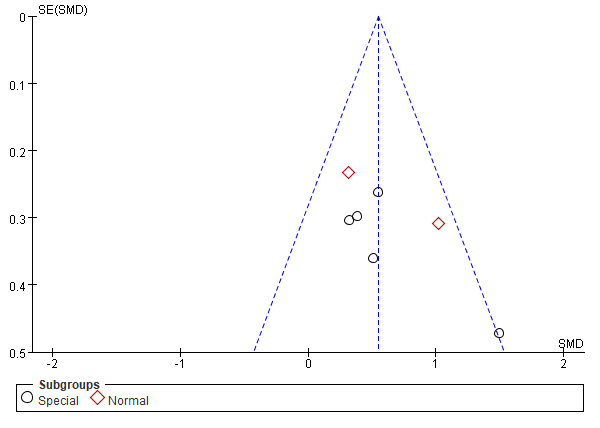


Disorder status


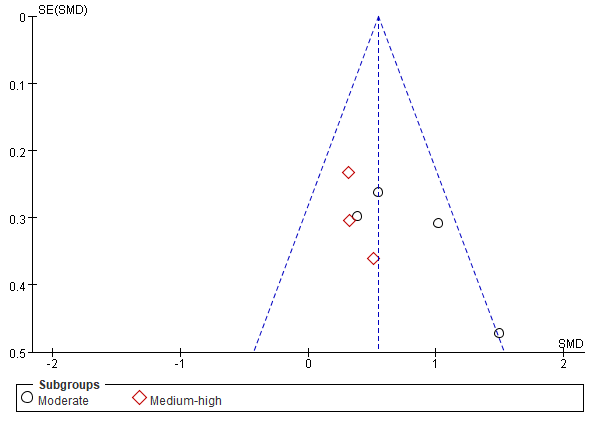


Exercise intensity


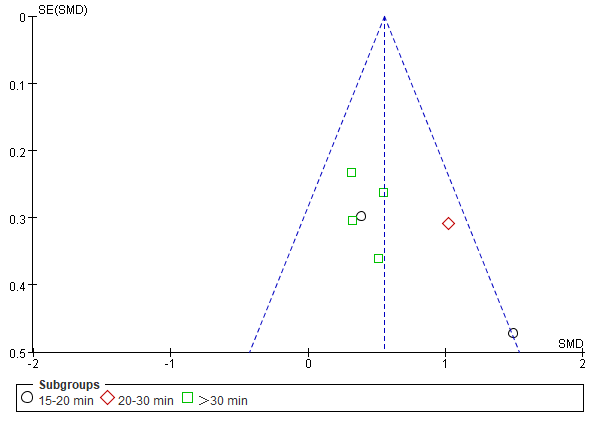


Intervention duration


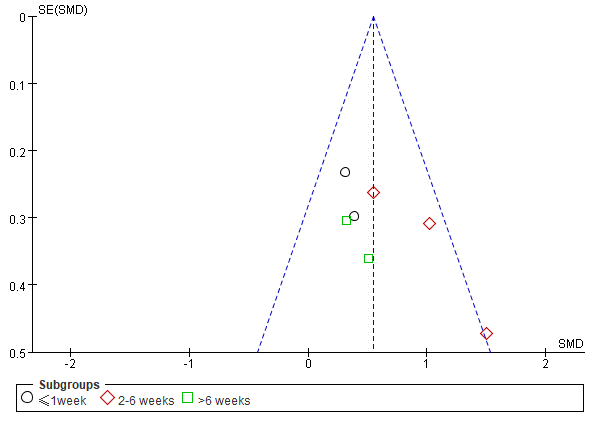


Intervention cycle

#### Working memory：


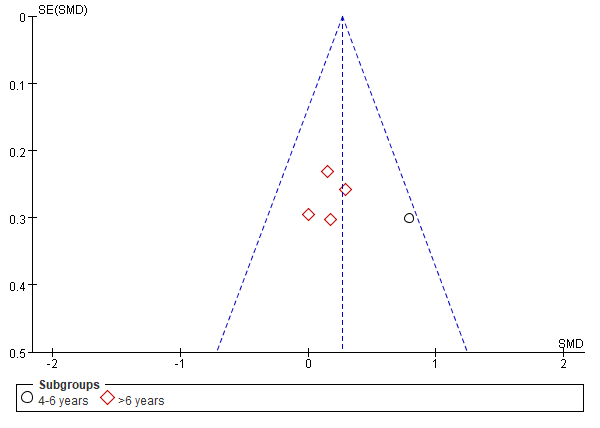


Age


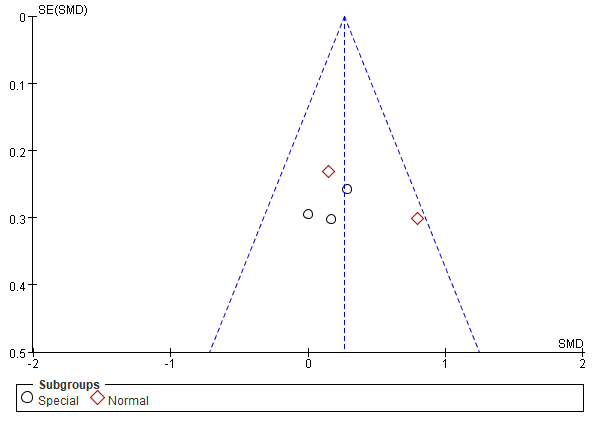


Disorder status


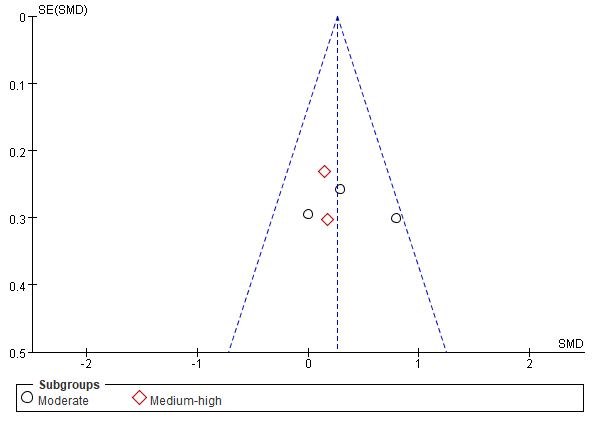


Exercise intensity


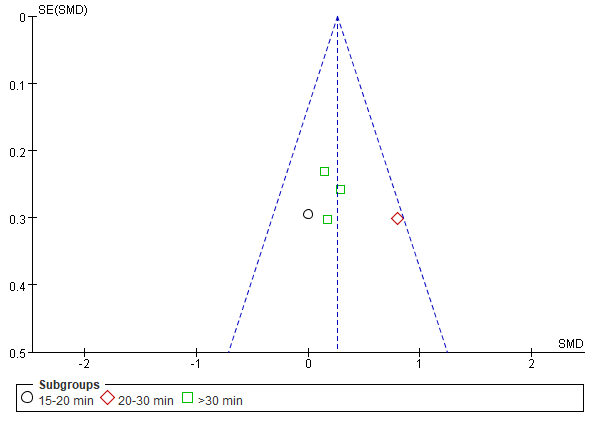


Intervention duration


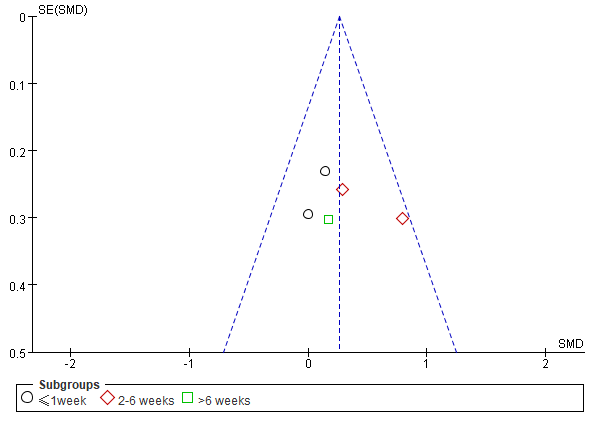


Intervention cycle
